# Synthesis of MR fingerprinting information from magnitude-only MR imaging data using a parallelized, multi network U-Net convolutional neural network

**DOI:** 10.3389/fradi.2024.1498411

**Published:** 2024-12-16

**Authors:** Kiaran P. McGee, Yi Sui, Robert J. Witte, Ananya Panda, Norbert G. Campeau, Thomaz R. Mostardeiro, Nahil Sobh, Umberto Ravaioli, Shuyue (Lucia) Zhang, Kianoush Falahkheirkhah, Nicholas B. Larson, Christopher G. Schwarz, Jeffrey L. Gunter

**Affiliations:** ^1^ Department of Radiology, Mayo Clinic, Rochester, MN, United States; ^2^ Department of Radiology, University of Iowa, Iowa City, IA, United States; ^3^ Department of Radiology, University of Texas Southwestern Medical Center, Dallas, TX, United States; ^4^ University of Illinois at Urbana-Champaign, Champaign, IL, United States; ^5^ Division of Biomedical Statistics and Informatics, Mayo Clinic, Rochester, MN, United States

**Keywords:** U-Net, convolutional neural network, magnetic resonance fingerprinting, MPRAGE, relaxometry

## Abstract

**Background:**

MR fingerprinting (MRF) is a novel method for quantitative assessment of *in vivo* MR relaxometry that has shown high precision and accuracy. However, the method requires data acquisition using customized, complex acquisition strategies and dedicated post processing methods thereby limiting its widespread application.

**Objective:**

To develop a deep learning (DL) network for synthesizing MRF signals from conventional magnitude-only MR imaging data and to compare the results to the actual MRF signal acquired.

**Methods:**

A U-Net DL network was developed to synthesize MRF signals from magnitude-only 3D *T*_1_-weighted brain MRI data acquired from 37 volunteers aged between 21 and 62 years of age. Network performance was evaluated by comparison of the relaxometry data (*T*_1_, *T*_2_) generated from dictionary matching of the deep learning synthesized and actual MRF data from 47 segmented anatomic regions. Clustered bootstrapping involving 10,000 bootstraps followed by calculation of the concordance correlation coefficient were performed for both *T*_1_ and *T*_2_ MRF data pairs. 95% confidence limits and the mean difference between true and DL relaxometry values were also calculated.

**Results:**

The concordance correlation coefficient (and 95% confidence limits) for *T*_1_ and *T*_2_ MRF data pairs over the 47 anatomic segments were 0.8793 (0.8136–0.9383) and 0.9078 (0.8981–0.9145) respectively. The mean difference (and 95% confidence limits) were 48.23 (23.0–77.3) s and 2.02 (−1.4 to 4.8) s.

**Conclusion:**

It is possible to synthesize MRF signals from MRI data using a DL network, thereby creating the potential for performing quantitative relaxometry assessment without the need for a dedicated MRF pulse sequence.

## Introduction

The power of MRI as a noninvasive diagnostic test is due not only to the range of soft tissue contrasts and functional information generated but more significantly to their correlation with anatomic and physiologic changes across a range of conditions and disease states (1). In the clinical setting, this versatility is utilized by executing several MR pulse sequences that provide multiple visualizations and quantitative data of the abnormality or disease in question. With the potential for each acquisition to last multiple minutes, MR examination times can range from tens of minutes to one or even two hours for specific imaging indications and number of anatomic regions covered. Thus, an MR exam represents a trade-off between allowing sufficient imaging time necessary to acquire the requisite MR data needed for diagnosis and the need to limit overall MR exam duration to ensure patient compliance, provide access to, and ensuring efficient and cost-effective use of an expansive and restricted imaging resource. Within this context, the demand to obtain additional imaging data—particularly that derived from multiple sequences—is limited.

Because MR image contrasts are a surrogate of the underlying and intrinsic relaxometry values of the tissue being imaged, it seems intuitive that quantitative assessment of these values would provide a more accurate and rapid diagnostic tool when compared to acquiring multiple MR data sets. Despite this, quantitative relaxometry methods have found limited clinical application due in part to their long acquisition times, the lack of multiparametric quantitation, and susceptibility to machine and environmental effects (2). Bobman et al. (3) described early approaches to multiparametric quantification and “synthetic” MR image generation in which a set of source images acquired with differing imaging parameters were used to generate quantitative relaxometry data. These data where then used as inputs into the Block equations for a given pulse sequence type. Although the approach demonstrated high precision and accuracy (4), long computing times impeded clinical introduction and widespread adoption.

Within the past decade there has been renewed interest in acquiring quantitative multiparametric imaging data, particularly from a single acquisition. One such approach, referred to as multi-dynamic multi-echo (MDME) or magnetic resonance imaging compilation (MAGIC) (5–7) involves acquiring multiple echo saturation recovery spin echo data from which relaxometry data are generated and then used as input to generate multiple synthetic MR images (contrasts). Another approach, first described by Ma et al. (8) and referred to as MR fingerprinting (MRF) involves the continuous repetition of a given MR imaging sequence, generating multiple 2D or 3D datasets of the object under interrogation. Unlike conventional steady-state MR imaging approaches in which parameters such as the pulse repetition rate (TR), echo time (TE), and the radio frequency (RF) excitation pulse flip angle (*α*) are held constant throughout the acquisition, MRF acquisition strategies rely upon varying multiple MR acquisition parameters according to a pre-determined history throughout the acquisition process. The result of this approach is to destroy the steady state signal, thereby making the acquired images non-diagnostic. However, given that the signal from a given pulse sequence can be described mathematically and that scan parameter values are known, the signal evolution of a given voxel can be generated for a chosen set of relaxometry parameters. If this process is repeated over a range of diagnostic relaxometry values, a so-called dictionary or library of signal evolutions can be generated (8–12). Comparison of the acquired signal evolution of a given voxel with the generated dictionary allows estimation of the actual relaxometry of the voxel in question by identifying the best match between the acquired and dictionary signals. Then, repetition of this process on a voxel-by-voxel basis allows for the quantitative and spatial resolution of these parameters. Additionally, once the spatial topography of various MR relaxometry parameters are quantified, multiple image contrasts can be synthesized by using these maps as inputs into the Block equation (13) describing the MR signal for the pulse sequence type and for the scan parameters of interest. Therefore, MRF provides a method for quantitative assessment of tissue relaxometry values, together with the ability to synthesize multiple MR image contrasts from a single acquisition.

Given the potential for MRF to address the limitations of conventional relaxometry approaches and the ability to synthesize multiple MR contrasts, MRF is an active area of research and development. However, limitations of this technology are the fact that it can only be used prospectively, thereby constraining its application to those subjects imaged since its inception (circa 2013) (8) and requires a dedicated pulse sequence and reconstruction pathway both of which are available only within research settings. Generation of a synthetic MRF signal obtained without the need for a dedicated MRF sequence has the potential to significantly expand this technology both in terms of prospective application but also by enabling its use retrospectively, thereby accessing the wealth of MR data acquired during the three-decade long period prior to the inception of the MRF technique.

Interest in the use of deep learning (DL) methods to solve a variety of challenges in MRI has increased significantly over the past several years given the promise of improved precision, accuracy and reduced computation times afforded by advanced DL models and graphical processing units (GPUs). Given the described limitations related to current MRF acquisition techniques and reconstruction methods, the development of a DL MRF method has the potential to address these and in doing so expand development and use of this promising technology. The purpose of this study is to report on the development and evaluation of a DL-based algorithm for synthesizing MRF data from a limited set of MR image information, specifically a rapidly acquired, magnitude only volumetric *T*_1_-weighted dataset. By contrast, this work distinguishes itself from existing DL approaches in MRF which are directed towards improvements in reconstruction accuracy and processing speed (14, 15).

## Materials and methods

### Subjects

This prospective study was approved by the lead author's Institutional Review Board (IRB), was HIPAA complaint and involved obtaining written informed consent from all subjects. Imaging data from 37 normal subjects were used in the study. Subject ages ranged from 21 to 62 years of age and included 10 males (minimum, maximum age = 24, 43 years) and 27 females (minimum, maximum age = 21, 62 years). There was no inclusion or exclusion criteria for subjects other than being able to successfully complete the MR imaging examination with recruitment being in response to internal, IRB approved research protocol advertising.

### Imaging protocol

All imaging was performed on a single 3T MR scanner (Signa Premier, GE Healthcare, Waukesha, WI). Each subject was imaged using the protocol listed in Table 1 that included a 3D MRF sequence described previously (16, 17) and multiple conventional MR imaging sequences (series). Each normal subject was scanned using a single 48 channel receive-only RF coil for signal reception.

**Table 1:** Standard imaging protocol used for normal subjects.

Pulse sequence	Prescription plane	Pulse sequence type	Acquisition mode	Field of view (cm)	Slice thickness (mm)	TR (ms)/TE (ms)/flip angle (degrees)	Excitations	Inversion time (ms)	Acquisition (*N_x_* × *N_y_* × *N_z_*)
Localizer		SE	3-plane		10	1,100/80/–	1	–	256 × 128
MRF	Axial	SSFP	3D	25.6	2	9.3//70^o^	1		256 × 256 × 256
Fat saturated *T*2 FLAIR	Sagittal	FSE	3D	24.0	1.4	6,300/105/	1	1,816	256 × 256 × 150
*T*1 CUBE DIR	Sagittal	FSE	3D	24.0	1.4	5,000/minimum/	0.5	2,497	256 × 256 × 150
MPRAGE	Sagittal	Gradient echo	3D	24.0	1.4	2,130/2.6/8^o^	1.0	900	192 × 192 × 150
DWI	Axial	EPI gradient echo	2D	24.0	4.0	1,700/minimum full/80^o^	2.0		128 × 128
*T*2 Spin Echo	Axial	Spin Echo	2D	24.0	4.0	3,000/102/111^o^	3.0		256 × 256

SE, spin echo, MRF, MR fingerprint, FLAIR, fluid attenuated inversion recovery, MPRAGE, magnetization prepared rapid acquisition gradient echo, DIR, double inversion recovery, DWI, diffusion weighted imaging.

MR image data were reconstructed by the host computer system of the MR scanner and stored in the DICOM imaging standard format while MRF reconstruction was performed offline using a proprietary Matlab (Mathworks, Natick, MA) software package provided by the University of Pisa (Pisa, Italy) on a Linux workstation equipped with two 8-core Intel Xeon Gold 6244 central processing unit and NVIDIA (NVIDIA Corporation, Santa Clara, CA) Tesla V100 graphical processing unit. Gomez et al. (9) have described the MRF processing pipeline that was used to process the raw MRF data into the first 15 singular value decomposition (SVD) coefficients of the temporal MRF signal (18). This compressed MRF signal was used to reconstruct quantitative parametric maps of *T*_1_, and *T*_2_ with relaxometry maps being generated in units of milliseconds (ms).

### DL network

DL involves training artificial neural networks to model complex patterns in data. These models, particularly convolutional neural networks (CNNs), have revolutionized various fields, including medical imaging, by enabling automated feature extraction and image classification. CNNs are particularly adept at recognizing spatial hierarchies in images through layers of convolutions, pooling, and nonlinear activations. In this study, we employed a U-Net architecture, a type of CNN originally designed for biomedical image segmentation (19).

MRF data consist of time-series signals which are projected onto the SVD space to reduce the dimensionality of the problem to a manageable level, as discussed by McGivney et al. (18). In this implementation, only the first four singular values (SV) are used, since higher order ones are found to contain very low signal and therefore only contribute to the noise component of the MRF SVD space. Phase renormalization is first applied by setting the imaginary component of the first SV (SV1) to zero. As a result, the real part of SV1 approximately resembles the spatial distribution of the proton density signal. Each complex SV is represented mathematically with a real and imaginary matrix associated with two distinct channels. Since SV1 is only real, the model establishes a multi-valued link between a single-channel MRI and seven-channel MRF information for the fours SV's.

**Figure 1:**
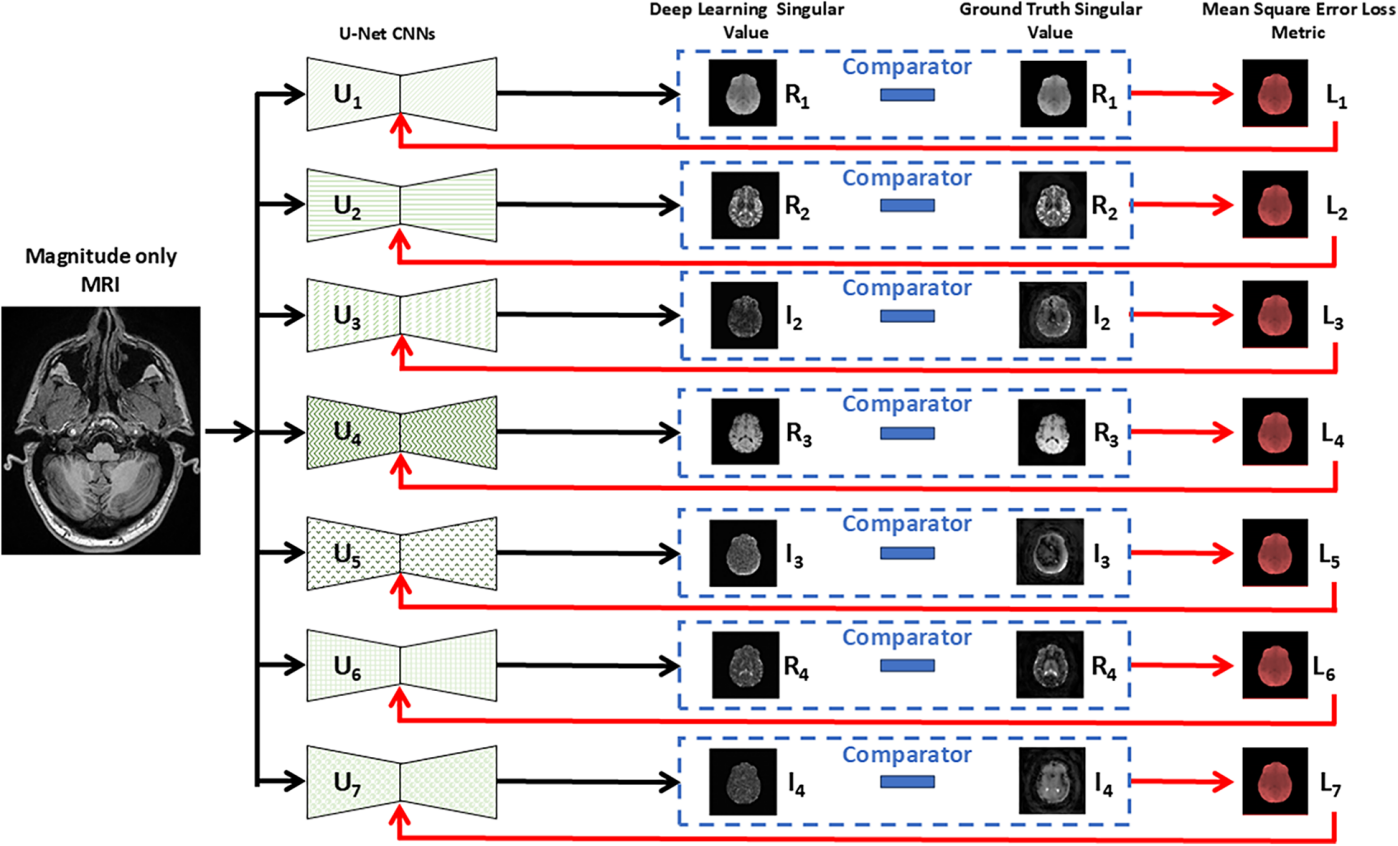
Schematic representation of seven parallel U-Net architectures utilized for regressing MRI input data. Each U-Net (*U*_1_ … *U*_7_) predicts a specific real or imaginary component of the singular value decomposition (SVD) from the multi-parametric MRF data. The predicted singular values are then compared (dashed box) with the ground truth continuous MRF singular values, and the mean squared error of each loss (*L*_1_ to *L*_7_) is employed to train iteratively each neural network, by updating the weights, for accurate reconstruction. Each singular value is complex and comprised of a real (*R*) and imaginary (*I*) component that is processed through separate U-Net convolutional neural networks (CNNs) except for the first singular value which was treated as being only real (*I*_1_ = 0) and therefore required only one CNN compared to higher order values that required two networks, one for the real and imaginary values.

**Figure 2:**
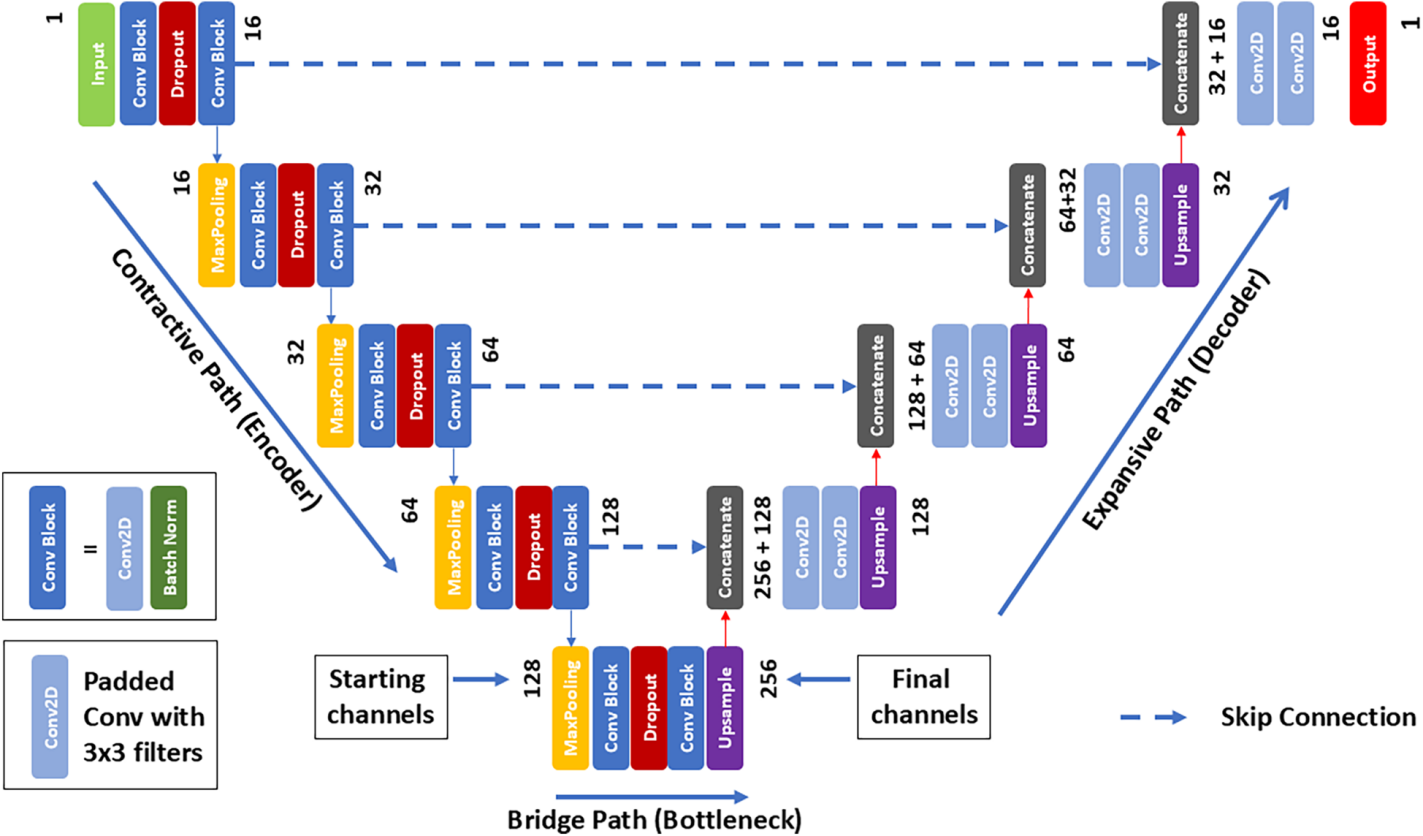
U-Net architecture for MRI image regression. The network begins with an input layer, proceeds through a contracting path on the left, characterized by convolutional and max pooling layers that increase in feature channels while incorporating dropout for regularization. The bottleneck at the center serves as a critical transition between the contracting and expansive paths, where up-sampling and concatenation with corresponding feature maps from the contracting path occur, followed by convolutions for detailed feature construction. The output layer, through a 1 × 1 convolution, translates the feature information into a continuous MRF space.

Figure 1 describes in block form the overall structure of the network which consists of seven identical DL networks. The network was designed to replicate the multichannel characteristics of the SVD MRF data with each network being considered an individually trainable channel. Each complex SV was considered a complex eigen value with each eigenvector being associated with two distinct channels, establishing a multiple-valued link between single-channel MRI and seven-channel MRF information. Each of the seven networks were trained simultaneously in the computational pipeline where the final composite network contains a total of 1,940,902 trainable parameters. The seven networks denoted *U*_1_ to *U*_7_ of Figure 1 are identical U-Net DL networks [fully convolutional DL network (20)] adapted for regression tasks (21). The overall architecture of each network is shown in Figure 2 which includes an enhancement achieved with batch normalization (22) and dropout layers to improve generalization and prevent overfitting (23). While these modifications are not novel in isolation, their inclusion in this adaptation of U-Net is intended to improve the robustness of the model in generalizing production of synthetic MR data from limited input MR images. Leveraging these established techniques serves to enhance the model's performance in predicting relaxometry values and synthesizing a wide range of image contrasts from a single acquisition.

By assigning distinct U-Net architectures to each SV, each individual network can be optimized to accurately reconstruct the specific component without interference or conflation. In a single network processing all components, the shared layers may unintentionally learn features that overlap or blend information from different SVs. This can dilute the specificity of the learned representations for each SV component. Instead, distinct networks for each component distribute the reconstruction tasks among independent networks, thereby favoring high fidelity reconstruction of synthetic MRF SVs. This is because errors in one component can propagate and degrade the overall quality of the reconstruction, affecting convergence. Multitask learning processes often struggle with interference between tasks when shared parameters optimize for diverse and potentially conflicting objectives. This is known as negative transfer and work in this area suggests that separating tasks can improve performance for problems with distinct characteristics. In our case these can be the different imaging modalities (e.g., proton density, *T*_1_, and *T*_2_), or simply the specific nature of the real and imaginary components of the SVs (24).

During the network training process, convergence is monitored by generating repeatedly synthetic MRF results from the MRI data sets reserved for verification. A running comparison is made of the synthetic MRF with the corresponding original MRF datasets which provide the ground truth and are never utilized in the network training process. Following the progression of the verification along with the training iterations ensures that convergence of both processes reaches acceptable levels of the error and that the downward trends show no significant overfitting behavior.

The U-Net is characterized by its U-shaped structure, which includes an encoder (contracting) path to capture context, and a decoder (expansive) path that enables precise localization. In between, the two paths are connected via a set of convolutional layers (bottleneck). Structurally, the network includes three consecutive pathways; a contractive path followed by a bridging and finally expansive path. 3D MRI data are input into each network and trained to generate each of the seven synthetic SVD outputs. While 3D data is used as input, each 3D data set is considered as a series of contiguous 2D inputs in which consecutive 2D inputs are fed into the network. Each slice follows the contracting path of the U-Net architecture, by application of two consecutive *convolution* steps with 16 different 3 × 3 *filters*. Each convolution is followed by a rectified linear unit (ReLU) and a batch normalization step generating a (256 × 256 × 16) feature block. Next, a 2 × 2 max pooling operation follows for down-sampling, in each of the separate 16 channels, resulting in a (128 × 128 × 16) block. The previous convolution scheme is repeated with a double number of 3 × 3 filters yielding now a (128 × 128 × 32) block. This overall down-sampling step is then repeated until a (16 × 16 × 256) feature block is obtained. The bridging or bottleneck step repeats the previous two consecutive convolution steps with the addition at the end of an up-sampling layer based on bilinear interpolation, which results in a (32 × 32 × 256) feature block.

After completion, the decoder expansive path begins with a concatenation step that combines the up-sampled block with the corresponding one obtained at the same level in the previous down-sampling path. Thus, the concatenation increases by 50% the number of channels. Then, two consecutive convolution steps follow as applied in the down-sampling path, but without the batch normalization step. At each up-sampling level, the same number of filters used in the corresponding down-sampling level is used ensuring that, after convolution, the same number of channels is reached. Finally, before climbing to the next level, another upscaling step is applied. The process is repeated until the top layer is reached, at which point the output contains a single channel. Each axial 2D slice of the 3D data are processed sequentially through the network until the entire 3D volume has been processed.

### Data preparation

Before being used for network training, both MRI and MRF data underwent several preprocessing procedures. The first involved selection of the appropriate MRI data. While multiple MRI contrasts were available as described in Table 1, only 3D magnitude prepared rapid gradient echo (MPRAGE) data was used as input MRI information due to its superior gray/white parenchymal differentiation (25) and to limit the complexity of the network by only accepting a single volumetric dataset as input. The second involved converting both data sets to the NIFTI format to ensure a single consistent format and image coordinate system. Thirdly, registration and resolution matching between the MRF SVD and MPRAGE data were performed to ensure equivalent spatial and geometric concordance of voxel pairs. Interpolation was performed on MPRAGE data producing equivalent 256 × 256 × 256 matrices with isotopic voxels of 1mm^3^. MPRAGE data underwent additional indexing to generate a 3D stack of axial slices. Registration was performed using the SimpleITK open-source package (26) and involved performing 3D rigid body registration and optimization based on mutual information optimization metric (26). Finally, normalization and skull stripping of both MRF and MRI data were performed. For MRI data, normalization involved rescaling the dynamic range of the voxel intensities to a minimum and maximum of 0 and 1 by dividing by the maximum voxel intensity of the volume. For MRF each real and imaginary SVD volume was similarly normalized between 0 and 1. Skull stripping was performed using binary masks of the whole brain generated as part of the tissue segmentation process described below. Both the segmentation and voxel-wise normalization steps were found to be necessary to ensure optimal model performance.

While interpolation is not necessarily considered as “augmentation”, it serves a similar purpose by artificially increasing the variability of training data or modifying data to fit specific needs. In this application, interpolation was applied to resample MRI images acquired with varying voxel sizes, to achieve a consistent resolution. Throughout this work MRF voxelization has served as the reference because it is generated on a regular grid as indicated above. Rigid affine transformations align the volumes but do not ensure voxel-to-voxel alignment of MRI to MRF data due to differences in voxel dimensions across different subjects. Registration and augmentation by resolution matching performed (resampling) between MRI an MRF data were therefore critical to ensure resolution consistency, effectively increasing the variety and quality of the training data.

### Network training and testing

To train the network, 16 slices were selected from the training data. Slices were randomly selected across subjects and location within the 3D imaging volume of each. Given that there were a total of 7,680 slices (30 subjects × 256 slices/subject) 480 iterations of the training phase were performed. The use of randomly sampled paired slices (MPRAGE, MRF) in this manner was necessary to address the need for substantial input data to attain the requisite precision and accuracy for quantitative analysis, amplifying the input volume by a factor of 256. While this strategy significantly augmented the dataset size, potential spatial correlations arising from contiguous slice acquisition were not accounted for in the model training.

To ensure convergence of the network, each synthetically generated SVD was rescaled using the ranges listed in Table 2. These values were generated from the maximum and minimum values of the ground truth MRF SVD values of the entire training set. Renormalization was applied to the SVD for each slice given that the network is designed as a 2D reconstruction network as opposed to the SVD of the entire 3D synthetic MRF volume.

**Table 2:** Minimum and maximum values of each component of the compressed ground truth MRF (i.e., acquired) data used for rescaling of the synthetic MRF singular values (SVs).

Singular value components	Minimum	Maximum
SV_1_ real	0.0	10.35
SV_2_ real	−3.09	0.65
SV_2_ imaginary	−1.83	1.88
SV_3_ real	−2.22	1.36
SV_3_ imaginary	−1.21	1.54
SV_4_ real	−1.95	0.90
SV_4_ imaginary	−1.16	1.30

These values are the maximum and minimum values obtained from the individual SVs of the 30 compressed MRF data used in training the network.

Quantification of network performance was achieved by comparison of the synthetic SVs and their ground truth counterparts. For each SV pair the batch mean squared error (MSE) in relation to the ground truth MRF data were calculated. Network weights were adjusted iteratively using an ADAM optimization algorithm (27), selected for its adaptive learning capabilities, and configured with a learning rate of 10^−4^. This process was repeated over numerous iterations, constituting an epoch (number of times network processes all data in the training set), with a total of 1,000 epochs executed for each network. Model performance was monitored, and the optimal weights corresponding to the lowest MSE throughout the training process were preserved. MSE is the standard choice of loss function for regression problems. As the differences between the ground truth and predicted values are squared, MSE tends to give more weights and thus be more sensitive to large errors.

### Estimation of relaxometry values

After training of the network, MPRAGE data from the five test subjects were input into the network to generate the four SVs for each. While the MPRAGE data was 3D, each dataset was treated as a stack of continuous 2D slices. As a result, 256 2D slices were input generating 256 × 4 complex SVs. The 256 2D SVs were combined to create a single 3D SV for each of the four complex values and rescaled to the global SV maximum and minimum values listed in Table 2. The 3D true and synthetic SVs were then input into the MRF dictionary matching algorithm (9, 28) using the software and hardware described above thereby generating 3D *T*_1_ and *T*_2_ data for both.

### Segmentation

MPRAGE derived anatomical regions of interest were generated using Statistical Parametric Mapping version 12 (SPM12) (SPM12: https://www.fil.ion.ucl.ac.uk/spm/) (29) with templates, settings and priors from the Mayo Clinic Adult Lifespan Template (MCALT: https://www.nitrc.org/projects/mcalt/). Subject specific segmentation maps were then applied to MRF derived relaxometry maps providing gray matter (GM), white matter (WM), cerebral spinal fluid (CSF) and whole brain (used for skull stripping described previously) segmentation maps. Additional segmentation was performed resulting in regional brain parcellations using the MCALT_ADIR122 atlas (https://www.nitrc.org/projects/mcalt/) (30) with Advanced Normalization Tools (31). In total, 47 individual regions were identified. For each region, the average relaxometry value (*T*_1_, *T*_2_) was calculated and used as input for statistical processing.

### Statistics

Region specific mean and standard deviation values were averaged for the five normal subjects for both true (i.e., acquired) MRF vs. DL MRF relaxometry values. To assess the degree of agreement between the relaxometry data pairs, the concordance correlation coefficient (CCC) was calculated. Prior to calculation of the CCC, clustered bootstrapping of data pairs (*T*_1_ true vs. *T*_1_ DL, *T*_2_ true vs. *T*_2_ DL) was performed using 10,000 bootstrapping operations to account for the multi-level nature of the data (multiple subjects and multiple correlated regions provided by the segmentation process). CCC values and 95% confidence intervals were calculated in addition to the mean difference between true and DL relaxometry values. All calculations were performed using the RStudio software package [Posit team (2023). RStudio: Integrated Development Environment for R. Posit Software, PBC, Boston, MA. http://www.posit.co/version 2023.12.0.369].

## Results

Table 3 lists mean and SD values averaged over the five normal subjects for both the true and DL derived *T*_1_ and *T*_2_ estimates for the 47 anatomical regions of interest. Overall, the DL estimates showed lower variance as measured by the average ratio of True to DL SD values across all subjects and regions (235 = 5 × 47 individual regions) and quantitated by the average ratio of 1.14 (minimum = 0.30, maximum = 2.19) and 1.79 (minimum = 0.35, maximum = 6.67) of the true to DL SD values. This is expected given the inherent smoothing nature of the DL process: The final layer of the neural network uses an activation function *tanh* which effectively compresses the data into the interval [−1, 1] with a low-pass filtering smooth function, as needed to maintain the stability of the training process.

**Table 3:** Mean and standard deviation values for the actual and deep learning (DL) generated relaxometry values for the five normal subjects for 47 anatomical regions of interest.

Segment	True *T*_1_		DL *T*_1_		True *T*_2_		DL *T*_2_	
	$\bar{T_{1}}$	$\bar{\sigma_{T_{1}}}$	$\bar{T_{1}}$	$\bar{\sigma_{T_{1}}}$	$\bar{T_{2}}$	$\bar{\sigma_{T_{2}}}$	$\bar{T_{2}}$	$\bar{\sigma_{T_{2}}}$
Precentral	1,219.05	188.74	1,179.95	157.26	53.57	18.95	52.06	6.01
Frontal	1,091.95	324.63	1,034.93	263.09	56.47	25.18	55.25	21.38
Rolandic operculum	1,157.36	337.79	1,087.97	242.20	56.98	48.13	52.55	23.03
Superior motor area	1,174.16	247.40	1,137.35	236.75	54.84	42.99	59.62	55.05
Olfactory	1,129.85	230.67	1,101.78	208.78	52.75	24.97	53.41	13.02
Frontal superior medial	1,240.88	174.22	1,117.40	181.21	57.17	11.41	56.41	9.52
Frontal medial orbital	792.91	64.11	802.86	67.88	43.58	5.92	44.97	5.43
Rectus	1,226.21	348.29	1,211.80	311.57	56.93	21.15	59.02	17.90
Insula	1,165.76	296.00	1,110.20	235.98	48.41	18.41	47.56	8.36
Cingulum	1,112.00	376.06	1,041.23	264.94	58.04	49.78	53.23	22.49
Hippocampus	915.58	84.70	897.35	48.87	33.42	6.04	34.26	2.21
Amygdala	1,225.21	248.49	1,173.07	198.07	54.70	23.71	53.82	7.24
Calcarine	1,158.93	371.97	1,104.69	318.70	80.25	74.91	74.68	54.98
Cuneus	1,229.30	175.10	1,161.40	221.10	71.47	16.24	70.73	17.15
Lingual	810.18	53.54	835.18	98.76	52.95	7.96	54.00	7.51
Occipital	1,234.78	344.30	1,164.70	276.76	72.91	39.83	72.21	25.97
Fusiform	1,316.22	466.33	1,287.05	420.25	114.33	130.23	99.89	82.17
Postcentral	1,196.08	248.40	1,131.24	212.94	52.20	19.75	52.50	15.07
Parietal	1,226.17	375.31	1,198.44	347.56	55.63	31.66	53.94	16.21
Supramarginal gyrus	1,251.48	237.10	1,220.97	176.74	54.35	39.23	53.88	11.69
Angular	1,338.31	320.69	1,260.07	268.09	61.54	28.85	58.94	14.76
Precuneus	1,182.13	330.25	1,116.01	280.77	53.70	44.31	49.44	20.05
Paracentral lobule	997.40	286.32	944.59	215.99	51.52	23.10	50.74	15.05
Caudate	1,240.88	174.22	1,117.40	181.21	57.17	11.41	56.41	9.52
Putamen	792.91	64.11	802.86	67.88	43.58	5.92	44.97	5.43
Pallidum	1,300.74	253.88	1,223.73	197.90	49.93	12.68	51.44	7.02
Thalamus	923.59	89.64	871.30	59.08	33.14	6.12	35.20	2.73
Heschl's gyrus	1,239.57	434.63	1,210.43	390.69	81.42	77.59	69.18	46.19
Temporal	1,221.05	265.70	1,120.69	221.14	47.17	17.75	46.21	9.04
Cerebellum	1,240.66	460.13	1,188.84	406.53	88.44	106.28	80.91	82.54
Vermis	1,225.46	161.34	1,188.85	288.09	63.29	26.21	67.66	35.23
Pons	822.00	55.10	860.70	106.77	47.36	9.55	49.60	8.62
Dorsal mesopontine	943.77	189.18	897.25	145.36	37.06	11.99	36.64	5.60
Entorhinal cortex	1,236.87	493.50	1,181.11	429.46	101.21	130.68	84.23	88.25
Para hippocampal	1,171.20	445.67	1,133.52	397.63	94.37	118.86	85.84	98.44
Cingulum posterior	1,243.10	385.92	1,166.64	314.87	64.51	59.76	59.39	37.00
Retrosplenial cortex	1,067.28	135.68	1,011.14	114.93	43.35	8.84	43.92	4.73
Frontal gray matter	1,171.88	280.44	1,103.91	253.17	59.50	23.20	59.50	13.21
Occipital gray matter	1,163.95	298.03	1,079.83	221.73	44.45	17.36	44.00	7.75
Parietal gray matter	1,294.66	399.55	1,184.95	335.35	68.82	53.80	61.55	27.98
Temporal gray matter	1,288.92	464.01	1,272.09	430.48	97.22	104.14	89.89	87.38
Cerebellum gray matter	1,165.18	383.41	1,104.97	326.07	63.64	40.17	60.18	31.17
Frontal white matter	1,123.70	303.51	1,076.94	263.71	58.80	28.40	57.65	17.88
Occipital white matter	1,239.66	178.45	1,165.70	197.19	61.94	15.95	61.23	11.72
Parietal white matter	817.22	60.55	831.06	86.89	44.08	7.71	45.84	6.91
Temporal white matter	1,043.98	195.88	1,006.65	180.79	39.94	18.30	39.44	14.05
Cerebellum white matter	1,287.52	321.54	1,270.22	317.31	59.32	52.13	58.62	32.41

The mean value $\left(\bar{T_{1}}\;\mathrm{and}\;\bar{T_{2}}\right)$ and the standard deviation of the mean $\left(\bar{\sigma_{T_{1}}}\;\mathrm{and}\;\bar{\sigma_{T_{2}}}\right)$ averaged across all volunteers for each region are displayed. True denotes the actual MRF derived relaxometry value while DL represents the DL equivalent.

**Figure 3:**
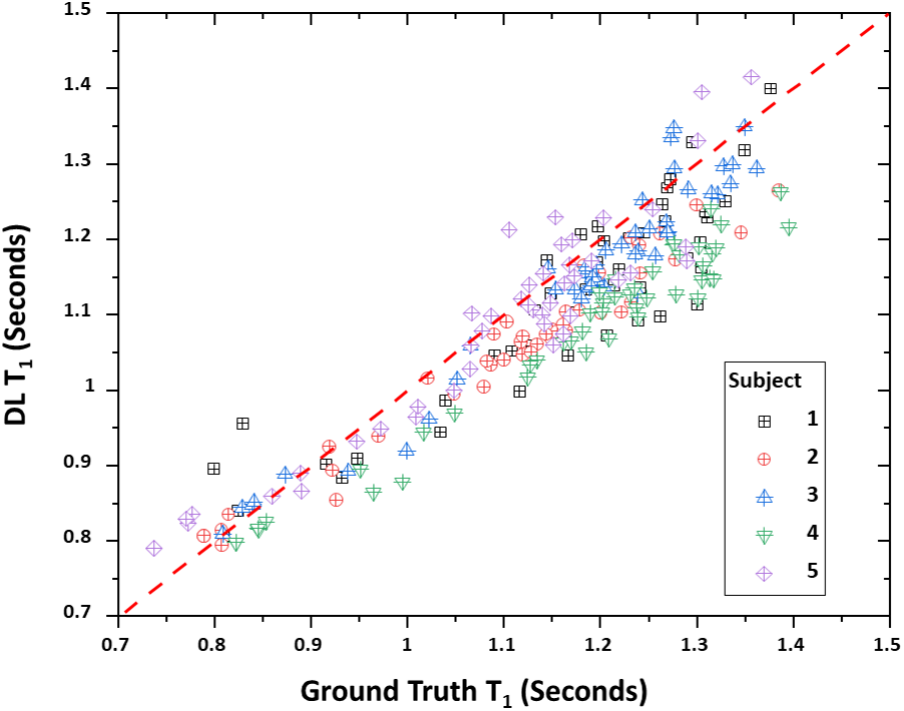
Scatter plot of true vs. deep learning (DL) *T*_1_ relaxometry values. A total of 235 points are shown with each point representing a given region (47 total) and subject (5 total).

**Figure 4:**
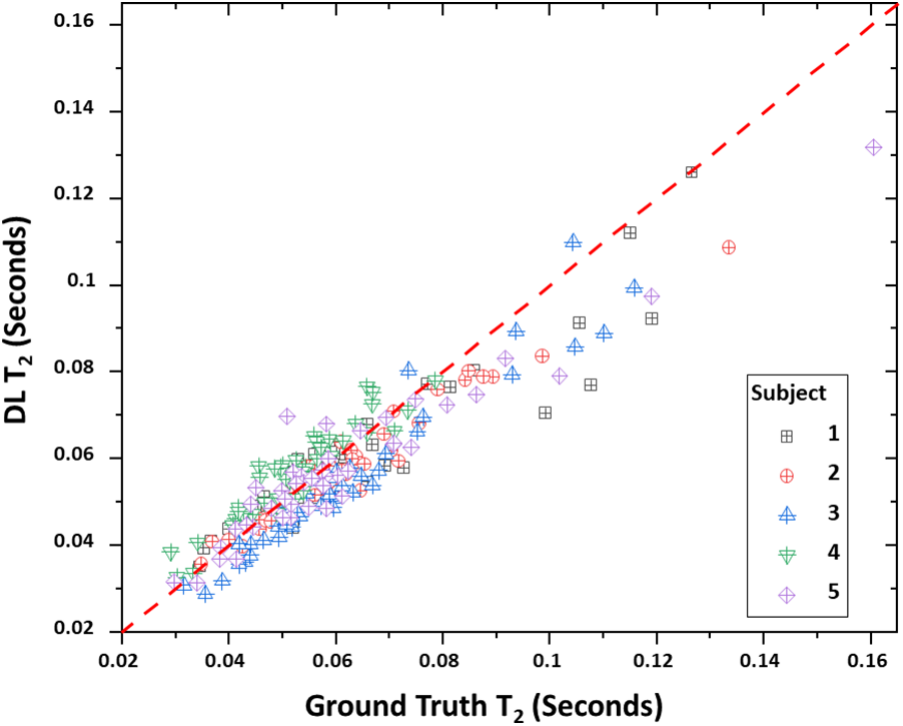
Scatter plot of true vs. deep learning (DL) *T*_2_ relaxometry values. *T*_1_ relaxometry values. A total of 235 points are shown with each point representing a given region (47 total) and subject (5 total).

Table 4 lists the bootstrap CCC values and 95% confidence intervals for both *T*_1_ and *T*_2_ true—DL data pairs. *T*_2_ values showed a slightly higher degree of correlation between the true and DL values compared to *T*_1_ (0.9078 vs. 0.8793). This is also reflected in the mean difference values with the mean differences being 48.23 and 2.02 ms for *T*_1_ and *T*_2_ respectively. The positive differences indicate an underestimation of DL relaxometry estimates compared to the acquired, i.e., true values. However, *T*_2_ estimates closer agreement due in part to the smaller absolute values and the fact that the 95% confidence intervals included zero difference. Figures 3, 4 show scatter plots of data pairs for *T*_1_ and *T*_2_ estimates for each region and subject (235 data pairs = 47 regions × 5 subjects) respectively and illustrate the bias, that is, the underestimation of relaxometry values estimated by the DL network.

**Table 4:** Concordance correlation coefficient (CCC) and 95% confidence intervals based on bootstrapping involving 10,000 bootstrap replicates.

Relaxometry parameter	CCC		$\bar{\mathrm{Tru}e_{T_{1,2}}-{\mathrm{DL}}_{T_{1,2}}}$	
	Value	95% confidence intervals	Value (ms)	95% confidence intervals
*T*_1_	0.8793	0.8136, 0.9383	48.23	23.0, 77.3
*T*_2_	0.9078	0.8981, 0.9145	2.02	−1.4, 4.8

True denotes the actual MRF derived relaxometry value while DL represents the deep learning equivalent.

**Figure 5:**
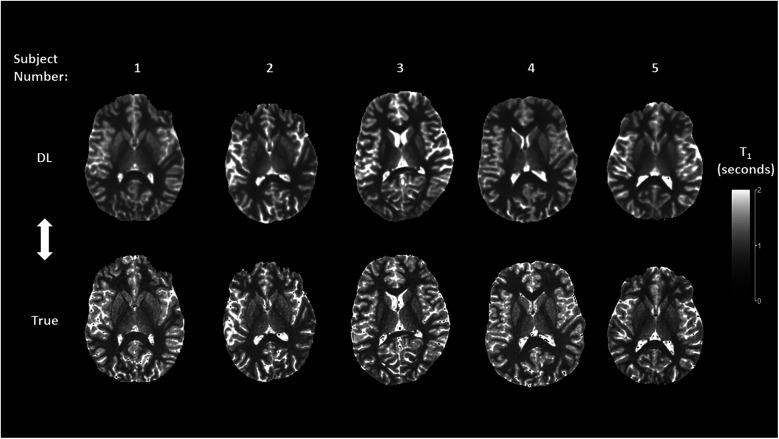
Mid-brain axial *T*_1_ relaxometry maps for the deep learning (DL) and true MRF reconstructions.

**Figure 6:**
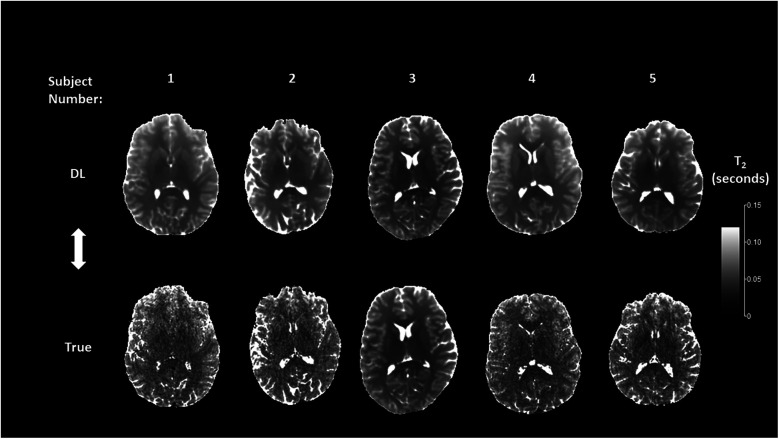
Mid-brain axial *T*_2_ relaxometry maps for the deep learning (DL) and true MRF reconstructions.

Figures 5, 6 show representative mid-brain axial slices of both true and DL reconstructed *T*_1_ and *T*_2_ relaxometry maps for the five normal subjects used for network testing. All data sets were preprocessed using an automated skull stripping algorithm and zeroing of non-brain (background) pixels and reconstructed to provide isotropic voxel dimensions (1 × 1 × 1 mm^3^). The same window and level settings were used for all *T*_1_ and *T*_2_ data (*T*_1_: window/level = 2/1 s, *T*_2_: window/level = 0.12/0.06 s). Both *T*_1_ and *T*_2_ DL maps were “smoother” in appearance which can be attributed to the inherent low-pass effect of the network noted previously while the true MRF data qualitatively exhibited lower signal-to-noise ratio (SNR) due to the apparent increase in image noise. The observed lower SNR of the true MRF relaxometry data is due to the relatively short acquisition time (∼4 min), high resolution, volumetric acquisition.

## Discussion

In this work we have developed a DL network for the purpose of generating a synthetic MRF signal from standard magnitude-only MR imaging data, in this case a *T*_1_-weighted (i.e., MPRAGE) 3D data of the brain of normal subjects. The potential for such a technique is that it provides the opportunity to generate quantitative relaxometry information from an MR examination that does not include an MRF as part of the original acquisition.

Given the complex acquisition strategies, computational requirements, and unique reconstruction methods of MRF, significant efforts are underway to integrate various DL based approaches to address these challenges. In general, these efforts can be categorized into those attempting to improve the precision and accuracy of quantitative relaxometry results, improve the computational efficiency of the data reconstruction process, or enhancement of specific MRF derived imaging applications. Efforts to improve the precision and accuracy of MRF-derived relaxometry values include using DL to increase the spatial resolution of MRF relaxometry data (32, 33), the accuracy and precision of quantitative relaxation values (34–39), decreasing MRF acquisition times (40), or replacing the dictionary matching process using DL (41). Similarly, DL has been used for MRF-related applications such as MRF chemical exchange saturation transfer (CEST) imaging (42–49), arterial spin labelling (50), improved anatomical mapping of disease processes (51–53), and MR spectroscopy (54). By contrast, the network described in this work has been designed with the specific intent of synthesizing an MRF signal from a previously acquired magnitude-only MR image data set. The implications of this approach are several fold; First, since the MRF technique was first reported in 2013 (8) there exists approximately 30 years of diagnostic MR image information that could benefit from this technology given that the first clinical MR imaging systems were developed in the early 1980s (55). Second, access to quantitative relaxometry information derived through a synthetic DL MRF could provide new insights into the development and progression of multiple disease processes by providing quantitative relaxometry information over time spans exceeding 50 years. Third, the described network demonstrates as a proof of concept the ability to derive quantitative information from an inherently qualitative (i.e., MRI) signal thereby opening new areas of investigation as well as DL methodologies for extracting quantitative metrics from inherently qualitative data.

Overall, as measured by the CCC, there was agreement between the DL-derived and actual MRF relaxometry values given their absolute values and 95% confidence intervals of 0.8793 and 0.8136–0.9383 for *T*_1_ and 0.9078 and 0.8981–0.9145 for *T*_2_. The authors have not attempted to assign a degree of agreement to these values given that there is disagreement between the interpretation of degree of agreement based on the absolute CCC value. For example. Akoglu (56) has described how CCC values can be interpreted as being similar to other correlation coefficients with values of <0.2 being assigned as poor and >0.8 as excellent. In contrast, Akoglu (56) also noted that other authors have indicated that poor agreement exists for values of <0.9 and substantial being within the range of 0.95–0.99. However, the data does indicate, as illustrated in Figures 1, 2 that strong agreement exists between both MRF approaches but that the degree of agreement is related to the absolute relaxometry value in question. This is particularly true for comparisons of *T*_1_ estimates across the rang of values (700–1,500 ms) while *T*_2_ estimates showed a decrease in DL-derived compared to actual MRF *T*_2_ estimates greater than 80 ms.

The discrepancies between relaxometry estimates are further quantified by the bootstrapped average (and 95% confidence intervals) of the difference between the true and DL-derived *T*_1_ and *T*_2_ estimates which were 48.23 ms (23.0–77.3 ms) and 2.02 ms (−1.4 to 4.8 ms) respectively. While the *T*_2_ confidence interval indicates that the mean difference includes zero, the *T*_1_ estimate did not indicating that, on average, the DL estimate of *T*_1_ was systematically less than the actual value. Previous comparison between calculated (i.e., MRF) estimates and National Institute of Standards and Technology (NIST)/International Society of Magnetic Resonance in Medicine (ISMRM) quantitative phantom (https://www.nist.gov/programs-projects/quantitative-mri) relaxometry values using the same MRF acquisition sequence have shown agreement for both *T*_1_ and *T*_2_ over clinically encountered relaxometry values (17). However, for both *T*_1_ and *T*_2_, the linear regression fit showed an intercept of 22.4 ms and 1.7 ms for the *T*_1_ and *T*_2_ values respectively with a positive intercept indicating an over estimation of the MRF-derived relaxometry value. Similar results were also reported by Buonincontri et al. (57) who performed a multicenter reproducibility study using a similar MRF sequence run on the same scanner manufacturer as used in this study in which both *T*_1_ and *T*_2_ derived relaxometry values were overestimated compared to the NIST stated values. Taken together, the underestimation of absolute *T*_1_ and *T*_2_ values by the DL network are compensated by the overestimation of these values by the actual MRF sequence therefore indicating overall accuracy and precision of the DL-derived values.

Comparison of the true vs. DL relaxometry maps as seen in Figures 5, 6 identify the overall smoothing of relaxometry maps generated from the DL MRF data when compared to the actual MRF relaxometry maps. This is to be expected given that DL in general and U-Net networks in particular are designed to find a local minimum of smoothly varying optimization functions and therefore are less susceptible to noise both in terms of signal value and spatial distribution. This is further quantified by comparison of the coefficient of variation (CoV) of the bootstrapped individual regional *T*_1_ and *T*_2_ estimates for both MRF approaches. For *T*_1_, the mean and range (minimum and maximum) of the CoV was 0.22 ms (0.066–0.399 ms) and 0.203 ms (0.054–0.364 ms) for the true and DL estimates across all 47 anatomic segments. Similarly, *T*_2_ estimates of the mean and range were 0.512 ms (0.136–1.291 ms) and 0.348 ms (0.065–1.048 ms) for the true and DL estimates respectively. Given the inherently noisy nature of the MRF data acquired in this work, the effect of smoothing introduced by the DL network was seen as an advantage thereby improving the precision of these estimates.

Our DL implementation differs from the standard U-Net architecture (20) in several ways. The batch normalization and the dropout layers have been added to avoid overfitting, which results in better prediction on untrained data. Seven distinct implementations of U-Net were trained independently and they were concatenated only at the final inference stage after convergence of the network weights. We plan to investigate in the future alternative implementations in which the networks are coupled during training, for example through weight sharing or weight pre-training strategies. For regression problems, as in this case, a linear activation function is commonly used for the output layer (21). However, this work adopted a tanh function which introduces nonlinearity in the prediction process for MRF data, yielding better convergence. Of note, the U-Net was originally designed for segmentation applications with a sigmoid activation function. Preliminary network configurations demonstrated poor convergence suggesting an ineffective activation function prompting the adoption of the tanh function. Future work will include assessment of other nonlinear activation functions.

A unique feature of the current network configuration was the use of a limited number of singular values and associated networks employed in generating the DL MRF. While the MRF compression algorithm generated a total of 14 complex SVs, initial investigation of all 14 indicated that most of the signal of the compressed MRF data was contained within the first four with the latter values contributing only noise to the system. Thus, the network was only trained on the initial four complex values except for the first SV in which the signal was considered to be only real (i.e., zero imaginary component). Zeroing of the imaginary component of the first SV was necessitated by the fact that their values were low, resulting in artifacts and a relatively large error function suggesting that the network was not optimized. Simply setting the imaginary component of the first SV eliminated this problem and resulted in rapid optimization, achieved in part by the optimization of seven vs. eight U-Net networks.

The results of this study suggest that it is possible to generate accurate *T*_1_- and *T*_2_-weighted relaxometry maps from a single rapidly acquired *T*_1_ dataset. *T*_1_ and *T*_2_ relaxometry data, in combination with proton density information makes possible synthetic MRI, allowing for creation of multiple additional contrasts typically used in clinical MRI. The MRF DL technique described herein has the potential to unlock hidden contrasts not typically seen by the eye on routine *T*_1_ images, and may enable calculation of *T*_1_, *T*_2_, fluid attenuated inversion recovery (FLAIR)-weighted images typically acquired on a traditional clinical MRI from a single acquisition. Furthermore, the approach provides the potential for adding multiparametric quantitative data without the need for additional imaging series, addressing concerns regarding increased MR examination times.

Recently Monga et al. (15) described developing trends in MRF and identified several emerging clinical applications including quantitative assessment of the heart, musculoskeletal, abdomen, brain and malignancies, specifically quantifying their response to radiation therapy. Collectively, they highlight the utility of MRF as a method for deriving quantitative MR biomarkers for multiple diseases and their related processes. However, a likely short-term application of MRF involves the assessment of intracranial diseases and tumors. This is due in part to the fact that *in vivo* feasibility was first demonstrated within the brain (8) but also because the brain is a relatively easy organ to image due to its overall spherical geometry, relative insensitivity to physiologic motion, and overall homogeneous tissue properties making correction of magnetic field inhomogeneities including both B_0_ and B_1_^+^ straightforward. Unsurprisingly, multiple authors have demonstrated the efficacy of MRF for diagnosing a range of disease processes and masses including assessment of mesial temporal lobe lesions associated with epilepsy (58), meningiomas (17), and multiple sclerosis (16). MRF is also providing increased clinical specificity particularly regarding further characterization and classification of both benign malignant tumors. For example, Badve et al. (59) demonstrated that MRF derived relaxometry values can differentiate solid tumor regions of lower grade gliomas from metastases and peritumoral regions of glioblastomas from lower grade gliomas. When MRF is combined with additional information, for example ^18^F PET-MR, it can be used to identify tumor grade and predict mutational status in gliomas (60), of inherent therapeutic significance. These data thus support the viability of the approach described in this work, particularly when applied to MRF of the brain.

The diversification of MRF applications to multiple organs and diseases highlights the clinical significance of quantitative relaxometry data in diagnostic MR imaging. The ability to synthesize an MRF signal from magnitude-only MR imaging data addresses a major limitation of this approach by allowing generation of this data without the associated MRF infrastructure (pulse sequence and reconstruction pipeline) and addressing clinical imaging constraints by not increasing overall examination times through the addition of extra pulse sequence acquisitions. Retrospective processing of existing MR data further points to the potential of this approach by creating additional opportunities for longitudinal studies that precede the arrival of MR fingerprinting.

The significance of this work is multifaceted. The proof-of-concept results presented highlight the transformative potential of DL to address current challenges in medical imaging that extend beyond the specific application of MRF. Also, since MRF is still not widely available nor integrated in routine clinical workflows, the development of this and other DL-based methods and tools, once appropriately trained and tested, have the potential to facilitate synthetic MRF information rapidly and inexpensively, thus contributing to efficiency and resource optimization. Finally, by demonstrating the feasibility and accuracy of this approach in normal subjects, the groundwork for extending these techniques to patients with various pathologies in the future has been established. As a first-of-its-kind method, this study has focused on establishing the feasibility and accuracy of generating synthetic MRF data from MRI. Unfortunately, benchmarking with other existing methods is not possible, due to the lack of prior approaches addressing this specific problem.

## Limitations

There are several limitations associated with this study. First, the DL network has been trained based on a single MRF pulse sequence and acquisition strategy on a single MR scanner and field strength. To address this, thereby increasing the generalizability of the approach, ongoing work is being performed to train and evaluate additional networks to create a synthetic MRF that can be described by a generalized scan parameter history that would accommodate various acquisition strategies, scan times and pulse sequences. This includes using MRF sequences from other MR scanner manufacturers and obtaining data from scanners at differing field strengths. Second, the network has only been trained on contiguous 2D data from the brains of normal subjects based on *T*_1_-weighted MR data thereby potentially reducing the sensitivity of the network to the *T*_2_ component of the synthetic MRF signal. It is important to note that, while heavily *T*_1_-weighted, the MPRAGE signal does include a *T*_2_ signal component (61) thereby influencing the learning phase of the network. Also, the incorporation of additional imaging data from a given subject such as *T*_2_-weighted or other contrast data sets greatly increases both the complexity of the network as well as the computation time, requiring additional computational resources. This also imposes a practical challenge of providing spatially registered data of equal resolution acquired at the same timepoint as input to the network which may not be available in a prospective clinical setting. Ongoing work is currently underway to train additional networks based on multiple MRI data inputs including *T*_2_-weighted data from patients referred to our clinical imaging practice and to input a single 3D volume into the network. We are therefore transitioning network development from a single stand-alone university server to national supercomputing resources, in particular the Delta GPU cluster at the National Center for Supercomputing Applications (delta.ncsa.illinois.edu). Finally, a small number of subjects were used for this study in all phases of the network development and training. However, we were still able to obtain excellent convergence by applying data augmentation. While the MRF data consists of a regular 3D grid of (256)^3^ voxels with 1 mm^3^ resolution, the available MRI scans are obtained with larger spacing between slices and the corresponding 2D grids, although regular, have usually lower resolution (larger grid spacing). The MRI data was carefully augmented by interpolation during the process of alignment with the MRF volume, which increased the effective distribution of MRI samples on a regular grid, used in training. Such augmentation techniques are widely used in machine learning to improve resolution and reduce model overfitting. Despite these limitations, the results indicate that a synthetic MRF signal can be generated from a single contrast MRI data set. We predict that additional network development and training will further increase the precision, accuracy and general applicability of the DL network.

## Conclusion

The results of this study support the hypothesis that MRF signals can be synthesized from conventional MR imaging data using a DL network. Overall agreement between the acquired and synthetic MRF signals were acceptable for both *T*_1_ and *T*_2_ derived relaxometry estimates for normal brain tissue at 3T. The work also demonstrates the potential to retrospectively analyze MR imaging information in the absence of an MRF signal, thereby enabling quantitative relaxometry to be performed on data acquired prior to the development of the MRF technique. Future work includes expanding the DL network capabilities to synthesize MRF data from multiple MR scanner manufacturers and to train additional networks on multiple *T*_1_-weighted and non-*T*_1_-weighted image contrasts.

## Data Availability

The datasets presented in this article are not readily available because original MR data is maintained and owned by Mayo Clinic. Requests to access the datasets should be directed to mcgee.kiaran@mayo.edu.
